# Pacific and Atlantic *Lepeophtheirus salmonis* (Krøyer, 1838) are allopatric subspecies: *Lepeophtheirus salmonis salmonis* and *L. salmonis oncorhynchi* subspecies novo

**DOI:** 10.1186/1471-2156-15-32

**Published:** 2014-03-14

**Authors:** Rasmus Skern-Mauritzen, Ole Torrissen, Kevin Alan Glover

**Affiliations:** 1 Institute of Marine Research, P.O. Box 1870, Nordnes. 5817, Bergen, Norway; 2 Sea Lice Research Centre, Department of Biology, University of Bergen, Bergen, Norway

**Keywords:** Hybrid fitness, COI, Subspecies, Sea lice, Salmon louse, Reproduction, Copepodids, Phylogenetics

## Abstract

**Background:**

The salmon louse *Lepeophtheirus salmonis* is a parasitic copepod that infects salmonids in the Pacific and Atlantic oceans. Although considered as a single species, morphological and biological differences have been reported between lice from the two oceans. Likewise, studies based on nucleotide sequencing have demonstrated that sequence differences between Atlantic and Pacific *L. salmonis* are highly significant, albeit smaller than the divergence observed between congeneric copepod species.

**Results:**

We demonstrated reproductive compatibility between *L. salmonis* from the two oceans and successfully established F2 hybrid strains using separate maternal lines from both the Pacific and Atlantic. The infection success for the F2 hybrid strains were similar to results typically observed for non hybrid lice strains in the rearing facility used. *Lepeophtheirus salmonis* COI and 16S sequences divergence between individuals from the Pacific and the Atlantic oceans was high compared to what may be expected within a copepod species and phylogenetic analysis showed that they consistently formed monophyletic clades representing their origin from the Pacific or Atlantic oceans.

**Conclusions:**

*Lepeophtheirus salmonis* from the Pacific and Atlantic oceans are reproductively compatible at least until adults at the F2 hybrid stage, and should not be regarded as separate species based on reproductive segregation or sequence divergence levels. Reported biological and genetic differences in *L. salmonis* seen in conjunction with the reported genetic diversity commonly observed between and within species demonstrate that Atlantic and Pacific *L. salmonis* should be regarded as two subspecies: *Lepeophtheirus salmonis salmonis* and *L. salmonis oncorhynchi* subsp. nov.

## Background

The salmon louse (*Lepeophtheirus salmonis* Krøyer, 1838) is a marine ectoparasitic copepod found on salmonids in the northern hemisphere. This parasite causes large economic losses in commercial cage-based salmon aquaculture [1,2], and has been causatively associated with declines in wild salmonid populations [3]. The latter of which is possibly linked with the fact that higher infestations of wild salmonids are typically observed in regions of intense commercial salmon farming [4-6]. Thus, *L. salmonis* represents both an economically and ecologically significant parasite in both the Pacific and Atlantic oceans which represent its natural distribution.

*Lepeophtheirus salmonis* displays a life cycle comprising eight stages: Two planktonic nauplius stages, an infective copepodid stage, two host-anchored chalimus stages and two motile preadult stages, before they ultimately molt into reproductive adults [7]. Thus, it displays considerable potential for dispersal, passively with ocean currents or while attached to its highly migratory hosts. This potential for dispersal has been confirmed by several recent genetics studies within the Atlantic [8-10] and Pacific [11] that have revealed panmixia or weak population genetic differentiation within each ocean basin. Once attached to its host, *L. salmonis* feeds on mucous and blood which causes physical damage, and may open wounds which can lead to osmoregulatory break-down and death in highly infected individuals [12,13]. At more modest infection levels, *L. salmonis* causes the host stress and initiates a cascade of gene regulation associated with stress response functions [14,15].

Several studies have investigated genetic differences between *L. salmonis* collected from the Pacific and Atlantic oceans. The first was based upon six microsatellite markers and revealed that 6% of the observed variation at these highly polymorphic loci was distributed among oceans [10]. A later analysis using the mtDNA gene cytochrome oxidase subunit 1 (COI) revealed 4.8-7.7% sequence divergence between Atlantic and Pacific *L. salmonis*[16]. By far the most comprehensive genetic comparison of *L. salmonis* from the Pacific and the Atlantic oceans has been conducted by Yazawa and colleagues [17]. Based upon the analysis of approximately 15 000 expressed sequence tags (ESTs), and sequence data covering the full mtDNA genome, these authors reported divergence between *L. salmonis* collected from these two oceans of 3.2% on average for nuclear genes, 7.1% for the entire mtDNA genome, 4.2% for ribosomal ribonucleic acid (rRNA) gene, and 6.1% for the COI gene. When the observed level of mtDNA divergence was compared with calibrated molecular clocks for copepods [18], it was concluded that the *L. salmonis* from the Pacific and Atlantic diverged approximately 2.5-11 million years ago. Additionally, reduced genetic variation has been observed among *L. salmonis* in the Pacific compared to the Atlantic leading Yazawa *et al.* to suggest that the species first established in the Atlantic and then in the Pacific following a limited introduction [17] when the Bering Strait opened approximately 5 million years ago [19].

Based upon the genetic diversity measures above, it has been proposed that *L. salmonis* from the Pacific and Atlantic may be regarded as separate forms of the same species [17] or even separate species [16]. Potentially in support of these suggestions is the fact that biological differences have also been identified between *L. salmonis* from these two oceans. These include reported differences in size [20,21], time of development [22,23] and possible differences in salinity tolerance [24,25]. Furthermore *L. salmonis* from the Pacific have been observed frequently naturally occurring [26,27] on three-spined sticklebacks (*Gasterosteus aculeatus*) while similar observations of common presence on non salmonid hosts have not been reported for *L. salmonis* in the Atlantic (but see [28,29]).

*Lepeophtheirus salmonis* have been investigated in laboratory studies for approximately three decades, and recently, extensive breeding facilities and rearing protocols have been refined making controlled crossing experiments possible [30]. Here, we report a study where we established two F2 generation hybrid strains of *L. salmonis* with genetic contribution from Pacific and Atlantic *L. salmonis* and address the questions: are they reproductively compatible and should they be regarded as one species?

## Methods

### *Lepeophtheirus salmonis* culturing and hybrid production

The rearing experiments described here were conducted at the Institute of Marine Research’s experimental facility in Bergen, Norway. This laboratory has a quarantine facility with permit issued by the Norwegian Food Safety Authority (NFSA) to conduct experiments on marine pathogens and includes chlorination of all waste-water to ensure that no pathogens are released to the natural environment. Lice were cultured using well-established rearing protocols for *L. salmonis*[30]. During the experiments Atlantic salmon were kept in running seawater and fed commercial fish feed *ad libitum*. This experiment was conducted in accordance with Norwegian legislation for use of animals in research, and was approved by the Norwegian Animal Research Authority (research permit nr. 2009/186329).

Lice were cultured by infecting Atlantic salmon (*Salmo salar*) with copepodids from Atlantic and Pacific strains of *L. salmonis*. The Atlantic strain (LsAtl) was established by mixing approximately equal numbers of copepodids from the previously described LsGulen and LsOslofjord strains [30]. The Pacific strain (LsPac) was established from copepodids derived from adult female *L. salmonis* collected at a commercial salmon farm located close to the town of Campbell River (British Columbia, Canada). The Pacific lice were transported to Norway in thermal flasks containing full strength salinity seawater. A permit to import these lice was obtained from the NFSA (Additional file 1). Unfertilized *L. salmonis* from the LsPac F1 and the LsAtl F2 generations were sorted according to sex at the pre-adult II stage and used immediately (LsAtl) or kept separate (LsPac) on previously uninfected fish until used in the crossing experiment.

**Figure 1:**
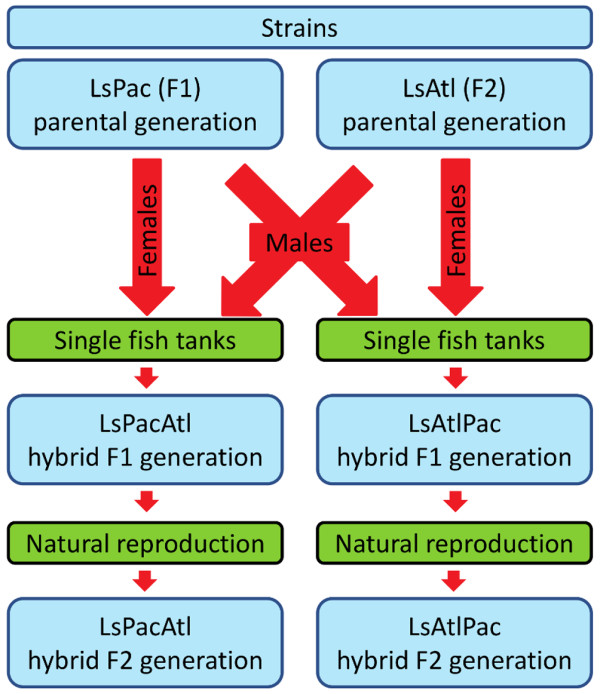
**Overall design of the breeding experiment.** The flow from the parental Atlantic and Pacific generations the F2 hybrid generations is illustrated schematically. For details refer to the main text.

To obtain hybrid strains we crossed LsPac females with LsAtl males and LsAtl females and LsPac males using a similar tank rearing system to that described by Hamre and Nilsen [31]. Briefly, each cross consisted of two (in one instance one) females and one male which were placed on a single uninfected Atlantic salmon that was isolated in its own tank. This allowed definite control of the parent material and offspring generation. As the unfertilized females were in different stages of development (pre-adult II and adults), the time required to obtain fertilized egg strings from the two hybrid strains were dissimilar. After 15 (LsPac females and LsAtl males) and 35 (LsAtl females and LsPac males) days, females bearing egg strings, and the males used to fertilize them in the single fish tanks, were harvested. These sampled adults were stored in individual tubes containing 100% ethanol, and were indexed so that both parents and egg strings could be subsequently matched. The egg strings from the sampled females were incubated in separate incubators after removal of approximately 3-5mm of both egg strings which was stored in 100% ethanol. The parents and the 3-5mm section of egg strings were genotyped (described below) to validate the crosses by parentage assignment before the resulting copepodids were used to infect previously uninfected Atlantic salmon to produce the LsAtlPac (copepodids from LsAtl females and LsPac males) and LsPacAtl (copepodids from LsPac females and LsAtl males) F1 hybrid strains. The F1 hybrid strains were reared separately in replicate fish tanks (Figure 1).

The F1 generation hybrids were allowed to develop, fertilize and reproduce naturally on Atlantic salmon in their respective tanks. Approximately 3 months after infection with F1 hybrid copepodids, eggstrings from F1 hybrid females that had been fertilized by F1 hybrid males were harvested and incubated in separate containers for LsAtlPac and LsPacAtl. Copepodids arising from these egg strings were thereafter used to infect groups of previously uninfected Atlantic salmon in order to establish the LsAtlPac F2 and LsPacAtl F2 hybrid generation in two replicate tanks for each strain. The resulting F2 hybrid generations were allowed to develop until the pre-adult stage to allow sex determination before the experiments were terminated. Numbers of adults contributing to each generation were recorded. In addition, the numbers of copepodids that were used to propagate the F2 generation was estimated by counting an aliquot, and the numbers of pre-adults harvested from the F2 generation upon termination of the experiment, were determined by counting and sex determining all the lice present.

### Genotype validation of F0 parents

In order to verify contributions of the individual pairs of parents to each set of hybrid egg strings for the F1 generation, both parents and an approximately 3-5mm section of each egg string was genotyped with microsatellite markers. DNA was extracted using a Qiagen DNeasy®96 Blood & Tissue Kit, and 16 microsatellite markers that have been recently used in a population genetic study of *L. salmonis* in the Atlantic ocean [8] were genotyped. These markers were amplified in three multiplex reactions. Multiplex 1; *LsalSTA1, LsalSTA2, LsalSTA4, LsalSTA5*[10], *LsNUIG14* adapted by [10], multiplex 2; *Lsal103EUVC, Lsal109EUVC, Lsal110EUVC, Lsal111EUVC*[11], *LsNUIG09*[32], multiplex 3; *Lsal104EUVC, Lsal105EUVC, Lsal106EUVC, Lsal108EUVC,*[11], *LsalSTA3*[10], *LsNUIG35B*[33]. Amplification conditions are given in Additional file 2. PCR fragments were separated on an ABI 3730XL sequencer and sized relative to the Applied Biosystem GeneScan™–500LIZ™ size standard. Alleles were scored using automatic binning implemented in the Genmapper software (V4.0). Allele profiles were manually inspected to validate that egg strings contained a maximum of 4 alleles per locus (i.e., two alleles from each parent if both were heterozygotes for the locus), and that all alleles matched with both parents where available.

### Comparing the Pacific and Atlantic 16S and COI sequences

To facilitate sequence comparison of 16S rRNA (16S) and cytochrome oxidase subunit I (COI) between the Pacific and Atlantic lice that were hybridized, the sequences were amplified and sequenced as described in Additional file 3. The obtained 16S sequences from the Pacific holotype specimen [GenBank: KF278676] and a representative female from the Atlantic founder generation for the F1 hybrids [GenBank:KF278677] were aligned with previously sequenced *L. salmonis* 16S sequences [9,17] from the Pacific [GenBank:EU288264-EU288330] and the Atlantic [GenBank:AY602770-AY602949]. The obtained COI sequences from the Pacific holotype specimen [GenBank:KF278676] and a representative female from the Atlantic founder generation for the F1 hybrids [GenBank:KF278677] were aligned with previously sequenced *L. salmonis* COI sequences [9,17] from the Pacific [GenBank:EU288201-EU288263] and the Atlantic [GenBank:AY602587-AY602766]. All sequences were aligned using CLC molecular workbench (6.8.2) at default settings and trimmed to the same length; 16S positions 84-879 (796 bp) and COI positions 121-1420 (1300 bp). All positions containing gaps and missing data were eliminated. Maximum likelihood phylogeny trees were constructed by MEGA5 [34] for each alignment using the Tamura-Nei model [35] and uniform rates for nucleotide substitution. Initial tree(s) for the heuristic search were obtained by applying the Neighbor-Joining method to a matrix of pairwise distances estimated using the Maximum Composite Likelihood (MCL) approach. Support for each of the two consensus trees were calculated by bootstrapping 1000 times.

## Results

The two founder strains (LsAtl and LsPac) were successfully established and subsequently used to produce the two F1 hybrid strains. These hybrid strains were thereafter propagated to produce two F2 hybrid strains. The number of adult females used to produce the copepodids founding each generation and the numbers of copepodids and resulting offspring for the hybrid strains are given in Tables 1 and 2. The low number of founding parents for the F1 hybrid strains (LsPacAtl and LsAtlPac) were caused by low fertilization success in the single fish tanks (each of which contained 2 females and a single male). In addition, some of the DNA samples taken from the egg strings from successfully fertilized females did not yield sufficient DNA to permit genotype validation of maternal and paternal contribution. These egg strings were not used. To establish the LsPacAtl F1 strain, the genetic contribution from both parents was validated with the allele profile of the egg strings. For the LsAtl F1 strain, the maternal origin was confirmed for all three pairs of egg strings used to establish the strain, but the paternal origin was only confirmed for one of these pairs. The last two pairs of egg strings shared the same father (i.e., were reared in the same single fish tank), but the father had been lost before sampling. Paternal contribution was however identical between these half-sibling families.

**Table 1:** The numbers of parents used to found each strain and generation

**Generation**	**Founding parents**
LsAtl F1	16 LsGulen and 11 LsOslofjord females
LsAtl F2	21 LsAtl F1 females
LsPac F1	9 LsPac F0 females (imported)
LsAtlPac F1*	3 LsAtl F2 females, 2 LsPac F1 males
LsAtlPac F2	19 LsAtlPac F1 females
LsPacAtl F1*	4 LsPac F1 females, 3 LsAtl F2 males
LsPacAtl F2	18 LsPacAtl F1 females

Male founders are only shown for LsAtlPac F1 and LsPacAtl F1 (marked with *) as these were the only crosses performed in single fish tanks with total control of paternal and maternal contribution.

**Table 2:** Biological characteristics for the F2 hybrid strains

**Source**	**Replicate**	**Copepodids**	**F2 males**	**F2 females**	**Infection success (%)**
LsPacAtl F1	1	1850	224	212	25
	2	1850	280	246	30
LsAtlPac F1	1	1000	93	81	17
	2	1000	158	124	28

The number of copepodids used to infect the hosts in the replicate tanks, and the numbers of males and females harvested upon termination of the F2 generation at the pre-adult stage are reported.

To tentatively estimate the fitness of the F2 hybrid strains, the numbers of copepodids used for infection of each replicate tank, and the numbers of pre-adult males and females harvested from the salmon, were recorded (Table 2). The infection success varied between 17 and 30%. Males were slightly overrepresented in all replicate tanks for both strains.

**Figure 2:**
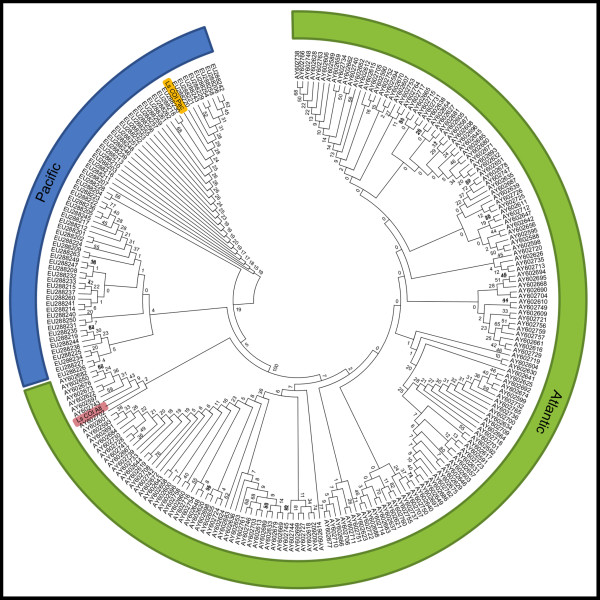
**Molecular Phylogeny of cytochrome oxidase subunit I (COI).** The evolutionary history of 245 nucleotide partial COI sequences was inferred by using the Maximum Likelihood method. The tree with the highest log likelihood (-11199) is shown. The analysis reveals two distinct phylogenetic clades with 100% bootstrap support corresponding to the Atlantic and Pacific samples respectively. The Pacific and Atlantic entries from the present study are highlighted in yellow and red. The tree is drawn to scale, with branch lengths measured in the number of substitutions per site. The Ls COI Pac sequence [GenBank:KF278676] was derived from the holotype speciemen (ZMUB91335). The Ls COI ATL sequence [GenBank:KF278677] was derived from a random female from the LsAtl strain.

**Figure 3:**
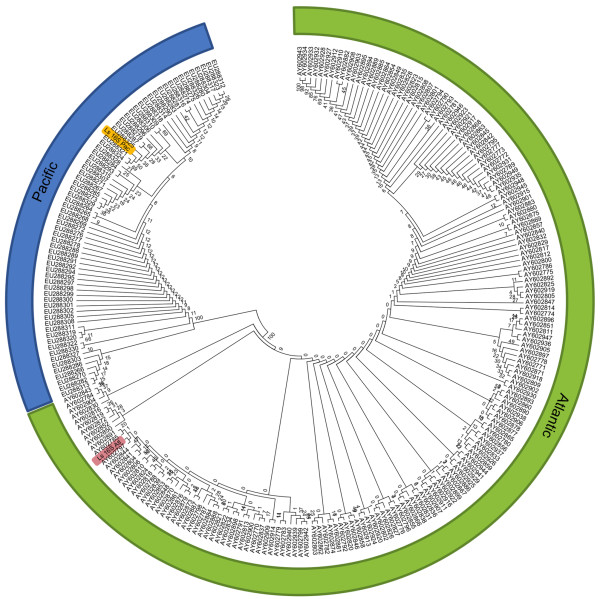
**Molecular Phylogeny of 16S ribosomal RNA.** The evolutionary history of 249 nucleotide partial 16S rRNA sequences was inferred by using the Maximum Likelihood method. The tree with the highest log likelihood (-2387) is shown. The analysis reveals two distinct clades with 100% bootstrap support corresponding to the Atlantic and Pacific samples respectively. The Pacific and Atlantic entries from the present study are highlighted in yellow and red. The tree is drawn to scale, with branch lengths measured in the number of substitutions per site. The Ls 16S Pac sequence [GenBank:KF278676] was derived from the holotype speciemen (ZMUB91335). The Ls 16S ATL sequence [GenBank:KF278677] was derived from a random female from the LsAtl strain.

The phylogenetic analysis showed that the Atlantic and Pacific sequences were found in separate clusters with 100% bootstrapping support for both 16S rRNA and cytochrome oxidase subunit I (Figures 2 and 3). The phylogenetic relationship within each ocean based on 16S and COI sequences has previously been the topic of detailed studies [9,17] and will not be discussed here.

### Nomenclatural acts

The electronic edition of this article conforms to the requirements of the amended International Code of Zoological Nomenclature. The ZooBank LSIDs (Life Science Identifiers) can be resolved and the associated information viewed through any standard web browser by appending the LSID to the prefix “http://zoobank.org/”. The LSID for this publication is: urn:lsid:zoobank.org:pub:8CABA147-FB3D-4DFA-AF37-7FFB74AFB454.

## Discussion

While the species concept and a single unequivocal definition of species remains a topic of discussion, most regard reproductive incompatibility between two groups of individuals as irrefutable evidence of separate species. Thus, reproductive incompatibility between *L. salmonis* from the Pacific and Atlantic would support the case for separating them into separate species as has been previously suggested based upon their COI genetic diversities [16]. In the present study, we successfully produced F1 and F2 hybrid *L. salmonis* strains using maternal lines from both the Pacific and the Atlantic. Previous studies of separate conspecific populations of copepods have shown that although defined as single species, the populations may or may not be reproductively compatible [36-38]. In studies of the intertidal copepod *Tigriopus californicus* it has been shown that “Dobzhansky-Muller incompatibilities” between strains, i.e. deleterious introductions of genetic variants into new genetic backgrounds, may result in reduced fitness in the F2 hybrids [39,40]. Although the present study was primarily designed to qualitatively investigate the reproductive compatibility of Atlantic and Pacific *L. salmonis*, egg string hatchability and survival between infection and the pre-adult stage was quantified for the two hybrid strains in the F2 generation. The observed values (Table 1) fall within the range of values for these parameters in this salmon louse rearing facility [30,41-43]. The infectivity and survival of Pacific *L. salmonis* reared in our facilities was not accurately quantified but did not appear to deviate from levels observed for Atlantic strains. Therefore, there are no data suggesting loss of fitness due to hybridization between Pacific and Atlantic *L. salmonis* up to and including the F2 hybrid generation. The genetic mtCOI divergence between *T. californicus* populations varies from 0.2% - 23% and the level of fitness reduction is correlated with the genetic divergence [44]. The genetic divergence between Atlantic and Pacific *L. salmonis* varies between 4.8 and 7.1% [16] and is thus limited when compared to the differences reported for *T. californicus*. This may suggest that outbreeding depression in hybrids between *L. salmonis* from the Pacific and Atlantic oceans may be expected to be limited if present, and could have gone undetected in the present study. It is therefore concluded that Pacific-Atlantic hybrid fitness should be accurately quantified in future studies, but that *L. salmonis* from the Pacific and Atlantic are reproductively compatible at least until the F2 hybrid generation. Therefore, our results do not support separating Atlantic and Pacific *L. salmonis* into separate species.

The COI gene has been sequenced for a large number of species and is currently being used for species identifications via DNA barcoding [45,46]. Looking at congeneric species (species within the same genus) across a wide range of taxa, 98% of species-pairs display COI divergence of 2% or more, and the average divergence across all taxa is 11.3% [46]. Within Crustacea, the average diversity among congeneric species has been estimated to 15.4% [46]. Looking specifically at copepods, congeneric COI sequence diversity was estimated between 13-22% [47]. The calculated divergence for COI between *L. salmonis* from the Pacific and Atlantic (4.8-7.7%) [16,17] is lower than what is typical for congeneric copepod species, but at the upper boundary of the reported variation of 1.3-7.9% within a crustacean species (i.e. between individuals of the same species) [48]. However, the congeneric calanoids *Calanus glacialis* and *C. finmarchicus*, with a reported COI sequence divergence of 20% [47], produce hybrids capable of successful reproduction [49]. Therefore, reproductive compatibility among Pacific and Atlantic *L. salmonis* may not be unexpected. Taken together, the reported sequence divergence of *L. salmonis* in the Pacific and the Atlantic oceans, which is well below the variations reported between congeneric copepods, and the evidence of reproductive compatibility with no apparent loss of fitness presented here, suggests that *L. salmonis* from the Pacific and Atlantic do not represent separate species.

While the genetic diversity within COI and 16S between *L. salmonis* from the Pacific and Atlantic is below that expected between species of copepods, it remains highly significant as illustrated by the phylogenetic analysis. This demonstrates minimal or non-existent genetic exchange between these allopatric *L. salmonis* components on a contemporary time-scale. This is in contrast to the very high level of gene-flow and lack of population differentiation reported among *L. salmonis* sampled from geographically distinct regions within each ocean [8,10,11,17]. Stable barcoding gene sequence divergences between morphologically and geographically defined subspecies, below the divergence level expected between congeneric species, has previously been used to confirm subspecies validity [50,51]. *Lepeophtheirus salmonis* from the Pacific and Atlantic exhibit morphological [20,21] and apparent biological differences [24,25,27] in addition to considerable genetic sequence divergence [17]. Despite these differences, Atlantic and Pacific *L. salmonis* are reproductively compatible and exhibit sequence divergence below the level typically observed between congeneric copepods and at the extreme upper boundary of the range found among conspecific crustaceans [48]. Therefore, we suggest that Atlantic and Pacific *L. salmonis* should be regarded as two subspecies: *Lepeophtheirus salmonis salmonis* and *Lepeophtheirus salmonis oncorhynchi* subsp. nov. urn:lsid:zoobank.org:pub:8CABA147-FB3D-4DFA-AF37-7FFB74AFB454.

Due to the interaction of salmon lice with farmed and wild salmonids in both the Pacific and Atlantic, *L. salmonis* represents both an ecologically and economically significant parasite. In accordance with this is the growing volume of scientific studies investigating this parasite. The differences between *L. salmonis* in the two oceans make it crucial to correctly assign scientific results to the correct component of the species, which is presently taxonomically impossible. The proposed division is therefore well reasoned and will facilitate appropriate taxonomic indication of origin in the future.

### Taxonomic summary of *Lepeophtheirus salmonis oncorhynchi* subsp. nov

Species: *Lepeophtheirus salmonis oncorhynchi* subsp. nov.

Etymology: ‘*oncorhynchi’* reflecting the evolutionary association with the salmonid genus *Oncorhynchus* in the Pacific Ocean

Descriptions: Johnson and Albright [20,22]

Holotype sequences: *Lepeophtheirus salmonis oncorhynchi* COI and 16S [GenBank:KF278676]

Museum specimens: Adult female ZMUB91335 (holotype, ethanol), adult male ZMUB91339 (allotype, karnovsky), paratype series, 3 specimens (ZMUB91336 – ZMUB91338, ethanol).

## Conclusions

*Lepeophtheirus salmonis* from the Pacific and the Atlantic oceans are reproductively compatible at least until the F2 hybrid generation, and evaluation of F2 hybrids does not indicate reduced fitness among the hybrids. The genetic divergences of COI and 16S between the Atlantic and the Pacific populations are below what one expects to find between crustacean species but high compared to what one may expect to find within a species. Taken together these results indicate that Pacific and Atlantic *L. salmonis* should not be regarded as separate species. However, morphological, genetic and indicated ecological differences are significant and salmon louse from the Pacific and the Atlantic oceans should therefore be regarded as two subspecies: *Lepeophtheirus salmonis salmonis* and *Lepeophtheirus salmonis oncorhynchi* subsp. nov. This is not only scientifically appropriate, but is of practical significance as this will facilitate taxonomic assignment of results obtained with either subspecies, something that has not been possible hitherto.

## Authors’ contributions

The study was conceived, designed and conducted by RSM, OT and KAG. The results were analyzed and the manuscript written by RSM and KAG. All authors read and approved the final manuscript.

## Supplementary Material

Additional file 1 **Certificate from the Norwegian Food Safety Authority (NFSA) for import of Pacific *L. salmonis *** **from the Pacific Ocean.** Click here for file

Additional file 2 PCR amplification conditions for the 16 microsatellite markers used to validate maternal and paternal contribution to the F1 hybrid crosses. Click here for file

Additional file 3 PCR amplification and sequencing of 16S and COI. Click here for file
