# Supplementary material for: Pacific and Atlantic Lepeophtheirus salmonis (Krøyer, 1838) are allopatric subspecies: Lepeophtheirus salmonis salmonis and L. salmonis oncorhynchi subspecies novo
Source: BMC Genet. 2014 Mar 14;15:32. doi: 10.1186/1471-2156-15-32 (PMC4007600; doi:10.1186/1471-2156-15-32)
Supplement: Additional file 1 — Certificate from the Norwegian Food Safety Authority (NFSA) for import of Pacific L. salmonis from the Pacific Ocean. [file 1471-2156-15-32-S1.pdf]

Mattilsynet

Application for import permission for consignments of animal by-products from third countries intended for research, analysis and/or test purposes. Not for human consumption.

This application form is to be used when applying for permission for import and use of by-products from third countries. The application form must be fully completed and presented to the Norwegian Food Safety Authority at least 21 days before planned date of entry.

Part I

Int. ref. no.

Applicant's details:

1. Name and full postal address of importing company or individual

Full name and title:

Institute of Marine Research (Havforskningsinstituttet)

Address, Postal Code / City

Nordnesgaten 50, P.O. Box 1870 Nordnes

5817 Bergen, Norway

Phone no. +47 55238500 / +47 55236371 / +47 90839556 | E-mail Olet@imr.no

Contact person: Ole Torrissen

2. Full postal address of destination premises if different from importers address:

Do the destination premises have any laboratory accreditation/recognized minimums standards? Yes

Please give details: See attached approval 2011/1923

Part II

Details of the animal by-product:

Identification of the animal by-product

Species | Nature of commodity | Treatment type if relevant NO

Sea Lice (Lepeophtheirus salmonis) | Live animals and egg strings & preserved samples in ethanol and formaldehyde

Origin of the by-product. Please state the origin of the material e.g. farms or laboratories:

Farm Lab Countryside/wild Abattoir commercial/processing plant other

Please specify:

Samples will be collected on commercial salmon farm (Marine Harvest), Campbell River, BC, Canada

If abattoir/commercial/processing plant, is the premises approved for export to the EU/EEA? If yes, state the EU approval number, if no is the premises approved under national legislation of the exporting country?

Please give details:

The farm is approved for export of salmon, but sea lice is not a commercial commodity

Quantity (weight) app. 10 g (net weight)	Number of packages 3	Type of packaging Thermoses filled with chilled sea water
Temperature of the product Ambient	Chilled 4 C	Frozen
Country of origin Canada ISO-code CA	Region of origin British Columbia	ISO-code BC
Place of loading Campbell River, BC	Planned date of arrival 29 Feb. 2011 at 16:00	
Means of transport Aeroplane<br>Identification: SAS (Courier)	Entry BIP in Norway Oslo, Gardermoen	

Part III

Intended use of the by-product

The product is intended for use as technical pharmaceutical cosmetic laboratory research other
Detailed description of intended use: The sea lice will be used in crossing experiments with Norwegian strains in order to identify mechanisms of drug resistance and evolutionary history.

Duration of research project:
The project (PrevenT) expires 31 Dec 2014

Plan for destruction of the animal by-product after use: The destruction will follow: "Internkontroll og prosedyrer ved våtlaboratoriene ved Havforskningsinstituttet i Bergen"

All documents (internal guidelines etc) must be enclosed. (Internkontrolldokumenter følger vedlagt)

Name and full postal address of the premises where the residues of the product following testing and any packaging materials will be incinerated: BIR Bedrift, Møllendalsveien 31, 5009 Bergen. (http://www.bir.no/birbedrift/Sider/Problemavfall.aspx)

Part IV

Applicants declaration

- **I am applying for a permission to import the above animal by-product under the provisions of the Norwegian legislation regulating import and by-products.**
- **I, the undersigned hereby declare that the information provided above is accurate to the best of my knowledge.**
- **I understand that if a permission is issued, I must comply in full to the conditions attached to such a licence including keeping appropriate records for three years and presenting these on request.**
- In particular, the consignment will not under any circumstances be used for human consumption or for feeding animals.

Place: Bergen Date: 7 feb 2012 Signature:____________________

Position held: Research program director

INSTITUTE OF MARINE RESEARCH
P.O.Box 1870 Nordnes - N-5817 Bergen - Norway

Part V

Decision for import and use of animal by-products for research, analyses and/or test-purposes

In pursuance to the provisions of :

- *Norwegian regulationFOR 1999-10-18 nr 1163: Forskrift om tilsyn og kontroll ved import og transitt mv. av animalske næringsmidler og produkter av animalsk opprinnelse mv. fra tredjeland* § 2.4.
- Norwegian regulation *FOR 2007-10-27 nr 1254: Forskrift om animalske biprodukter som ikke er beregnet på konsum.* §12. and
- REGULATION (EC) No 1774/2002 of THE EUROPEAN PARLIAMENT AND OF THE COUNCIL of 3 October 2002 laying down health rules concerning animal by-products not intended for human consumption Article 3.2 and article 23, 1.(a).
- Council Directive 97/78 of 18 December 1997 laying down the principles governing the organisation of veterinary checks on products entering the Community from third countries.

the Norwegian Food Safety Authority/ Border Inspection Post Oslo Airport Gardermoen, Norway

X **grants import-authorisation** ~~**does not grant import authorisation**~~

for the animal by-products described in part 2 to be used for research purposes described in part 3 of this application.

This decision may be appealed to the Regional Food Safety Authority in accordance with the provisions in the Norwegian Administration Act.
The appeal must be made within three weeks after receiving this decision. An appeal should be submitted to the regional office via the above mentioned Border Inspection Post.

07.02.2012

Date: ~~**24.05.2011**~~

Signature and official stamp

OSLO · VETERINARY BORDER CONTROL

Place:

Border Veterinary, Oslo Airpport

Gardermoen, Norway
