# Supplementary material for: Pacific and Atlantic Lepeophtheirus salmonis (Krøyer, 1838) are allopatric subspecies: Lepeophtheirus salmonis salmonis and L. salmonis oncorhynchi subspecies novo
Source: BMC Genet. 2014 Mar 14;15:32. doi: 10.1186/1471-2156-15-32 (PMC4007600; doi:10.1186/1471-2156-15-32)
Supplement: Additional file 2 — PCR amplification conditions for the 16 microsatellite markers used to validate maternal and paternal contribution to the F1 hybrid crosses. [file 1471-2156-15-32-S2.doc]

# Additional file 2.

## Multiplex1

|  | Concentration | | Reaction | | Volum (ul) | Volum (ul) |
| --- | --- | --- | --- | --- | --- | --- |
|  |  |  |  |  | 1 | 100 |
|  |  |  |  |  | Sample | Sample |
| **DNA** | 16,6 | ng/ul |  |  | 2 | 200 |
| **Buffer Go Taq** | 5 | x | 1 | x | 2 | 200 |
| **MgCl_2_** | 25 | mM | 2,25 | mM | 1 | 100 |
| **dNTP** | 10 | mM | 0,3 | mM | 0,3 | 30 |
| **GoTaq pol** | 5 | U | 0,75 | U | 0,1 | 10 |
| **STA1-F** | 10 | μM | 0,1 | μM | 0,1 | 10 |
| **STA1-R** | 10 | μM | 0,1 | μM | 0,1 | 10 |
| **STA2-F** | 10 | μM | 0,1 | μM | 0,1 | 10 |
| **STA2-R** | 10 | μM | 0,1 | μM | 0,1 | 10 |
| **STA4-F** | 10 | μM | 0,1 | μM | 0,1 | 10 |
| **STA4-R** | 10 | μM | 0,1 | μM | 0,1 | 10 |
| **STA5-F** | 10 | μM | 0,1 | μM | 0,1 | 10 |
| **STA5-R** | 10 | μM | 0,1 | μM | 0,1 | 10 |
| **LUIG14-F** | 10 | μM | 0,5 | μM | 0,5 | 50 |
| **LUIG14-R** | 10 | μM | 0,5 | μM | 0,5 | 50 |
| **H_2_O** |  |  |  |  | 2,8 | 280 |
| **Total** |  |  |  |  | 10 | 1000 |

PCR conditions: 95°C for 1 min; 30 cycles of 95°C for 30 s, 58°C for 30 s, 72°C for 30 s; 72°C for 2 min; 4°C∞

ABI dilution: 118 µl Millipore water + 2 µl PCR product (1/60)

Total volume pr. sample: 10 µl

Number of samples: 100

## Multiplex 2

|  | Concentration | | Reaction | | Volume (ul) | Volume (ul) |
| --- | --- | --- | --- | --- | --- | --- |
|  |  |  |  |  | 1 | 100 |
|  |  |  |  |  | Sample | Sample |
| **DNA** | 25 | ng/ul |  |  | 2 | 200 |
| **Buffer Go Taq** | 5 | x | 1 | x | 2 | 200 |
| **MgCl_2_** | 25 | mM | 2,5 | mM | 1 | 100 |
| **dNTP** | 10 | mM | 0,3 | mM | 0,3 | 30 |
| **GoTaq pol** | 5 | U | 0,75 | U | 0,1 | 10 |
| **LS-3 F** | 10 | μM | 0,1 | μM | 0,01 | 0 |
| **LS-3 R** | 10 | μM | 0,1 | μM | 0,01 | 0 |
| **LS-9 F** | 10 | μM | 0,1 | μM | 0,01 | 0 |
| **LS-9 R** | 10 | μM | 0,1 | μM | 0,01 | 0 |
| **LS-10 F** | 10 | μM | 0,07 | μM | 0,007 | 0 |
| **LS-10 R** | 10 | μM | 0,07 | μM | 0,007 | 0 |
| **LS-11 F** | 10 | μM | 0,1 | μM | 0,01 | 0 |
| **LS-11 R** | 10 | μM | 0,1 | μM | 0,01 | 0 |
| **NUIG-9 F** | 10 | μM | 0,25 | μM | 0,025 | 0 |
| **NUIG-9 R** | 10 | μM | 0,25 | μM | 0,025 | 0 |
| **H_2_O** |  |  |  |  | 4,476 | 447,6 |
| **Total** |  |  |  |  | 10 | 987,6 |

PCR conditions: 95°C for 1 min; 30 cycles of 95°C for 30 s, 58°C for 30 s, 72°C for 30 s; 72°C for 2 min; 4°C∞

ABI dilution: 118 µl Millipore water + 2 µl PCR product (1/60)

Total volume pr. sample: 10 µl

Number of samples: 100

## Multiplex 3

|  | Concetration | | Reaction | | Volume (ul) | Volume (ul) |
| --- | --- | --- | --- | --- | --- | --- |
|  |  |  |  |  | 1 | 100 |
|  |  |  |  |  | Sample | Sample |
| **DNA** | 25 | ng/ul |  |  | 2 | 200 |
| **Buffer Go Taq** | 5 | x | 1 | x | 2 | 200 |
| **MgCl_2_** | 25 | mM | 2,5 | mM | 1 | 100 |
| **dNTP** | 10 | mM | 0,3 | mM | 0,3 | 30 |
| **GoTaq pol** | 5 | U | 0,75 | U | 0,1 | 0 |
| **LS-4 F** | 10 | μM | 0,1 | μM | 1 | 0 |
| **LS-4 R** | 10 | μM | 0,1 | μM | 1 | 0 |
| **LS-5 F** | 10 | μM | 0,08 | μM | 0,8 | 0 |
| **LS-5 R** | 10 | μM | 0,08 | μM | 0,8 | 0 |
| **LS-6 F** | 10 | μM | 0,15 | μM | 1,5 | 0 |
| **LS-6 R** | 10 | μM | 0,15 | μM | 1,5 | 0 |
| **LS-8 F** | 10 | μM | 0,15 | μM | 1,5 | 0 |
| **LS-8 R** | 10 | μM | 0,15 | μM | 1,5 | 0 |
| **NUIG-35 F** | 10 | μM | 0,08 | μM | 0,8 | 0 |
| **NUIG-35 R** | 10 | μM | 0,08 | μM | 0,8 | 0 |
| **STA-3 F** | 10 | μM | 0,15 | μM | 1,5 | 0 |
| **STA-3 R** | 10 | μM | 0,15 | μM | 1,5 | 0 |
| **H_2_O** |  |  |  |  | -9,6 | -960 |
| **Total** |  |  |  |  | 10 | -430 |

PCR conditions: 95°C for 1 min; 30 cycles of 95°C for 30 s, 58°C for 30 s, 72°C for 30 s; 72°C for 2 min; 4°C∞

ABI dilution: 118 µl Millipore water + 2 µl PCR product (1/60)

Total volume pr. sample: 10 µl

Number of samples: 100
