# Supplementary material for: Pacific and Atlantic Lepeophtheirus salmonis (Krøyer, 1838) are allopatric subspecies: Lepeophtheirus salmonis salmonis and L. salmonis oncorhynchi subspecies novo
Source: BMC Genet. 2014 Mar 14;15:32. doi: 10.1186/1471-2156-15-32 (PMC4007600; doi:10.1186/1471-2156-15-32)
Supplement: Additional file 3 — PCR amplification and sequencing of 16S and COI. [file 1471-2156-15-32-S3.doc]

Additional file 3.

To obtain 16S rRNA (16S) and cytochrome oxidase subunit I (COI) sequence from Pacific and Atlantic lice used in the hybridization experiment the sequences were amplified by PCRs and the resulting fragments were sequenced by sanger sequencing.

Pacific *L. salmonis* DNA was obtained from an eggstring from the Pacific female *L. salmonis* *oncorhynchi* sp. nov. holotype (ZMUB91335) originating from the parental generation of the F1 hybrids. Atlantic L. salmonis DNA was obtained from a randomly selected Atlantic female from the parental generation of the F1 hybrids. The DNA was isolated using Quiagen DNeasy Blood and Tissue kit following the manufacturer’s instructions. PCR reactions were performed using 1.0μL template using the PCR setup outlined in Table 1-3 and the primers listed in Table 4. The PCR ran for 40 cycles as follows: 30s of denaturation at 95°C, 30s of annealing at the temperature specified in Table 1-3, and 2min of extension at 72°C. The products were evaluated by standard gel electrophoresis to confirm amplicon size.

The resulting amplicons were then sequenced by Sanger sequencing using BigDye 2.0 Chemistry (Applied Biosystems) and the primers specified in Tables 1-3. The sequences were imported and quality trimmed in CLC Workbench 6.8.2 using standard settings and contigs were subsequently constructed using the same software.

Table 1: LsCOI PCR and sequencing. The primers and annealing conditions are given in rows marked PCR #. The sequencing primers are given in the rows marked Seq. primers. The Atl DNA was purified from a random female from the LsAtl strain. The pacific DNA was purified from eggstrings from the holotype speciemen (ZMUB91335).

| PCR | Fwd | Rev | Anneal | Amplicon | template |
| --- | --- | --- | --- | --- | --- |
| Atlantic | | | | | |
| PCR 1 | LsCOI_Fwd | LsCOI_Rev | 55 | 1769 bp | Atl. DNA |
| PCR 2 | LsCOI_Fwd | COI_seq_R | 55 | 733 bp | Atl. DNA |
| PCR 3 | COI_seq_F | Ls_COI_Rev | 55 | 1128 bp | Atl. DNA |
| Seq. primers | LsCOI_Fwd, COI_seq_F, COI_seq_rev2, COI_seq_fwd2, LsCOI_Rev, COI_seq_R | | | | |
|  |  | | | | |
| Pacific | | | | | |
| PCR 4 | LsCOI_Fwd | LsCOI_Rev | 55 | 1769 bp | Pacific DNA |
| PCR 5 | LsCOI_Fwd | COI_seq_R | 55 | 733 bp | Pacific DNA |
| PCR 6 | COI_seq_F | Ls_COI_Rev | 55 | 1128 bp | Pacific DNA |
| Seq. primers | LsCOI_Fwd, COI_seq_F, COI_seq_rev2, COI_seq_fwd2, LsCOI_Rev, COI_seq_R | | | | |

Table 2: Ls16S PCR and sequencing. The primers and annealing conditions are given in rows marked PCR #. The sequencing primers are given in the rows marked Seq. primers. The Atl DNA was purified from a random female from the LsAtl strain. The pacific DNA was purified from eggstrings from the holotype speciemen (ZMUB91335).

| PCR | Fwd | Rev | Anneal | Amplicon | template |
| --- | --- | --- | --- | --- | --- |
| Atlantic | | | | | |
| PCR 1 | Ls_16S_Fwd | Ls_16S_Rev | 55 | 1358 | Atl. DNA |
| PCR 2 | Ls_16S_Seq_F | Ls_16S_Rev | 55 | 764 | Atl. DNA |
| PCR 3 | Ls_16S_Fwd | Ls_16S_Seq_R | 55 | 796 | Atl. DNA |
| Seq. primers | Ls_16S_Seq_F, Ls_16s_Rev, Ls_16S_Fwd, Ls_16S_Seq_R | | | | PCR prod |
| Pacific | | | | | |
| PCR 1 | Ls_16S_Fwd | Ls_16S_Rev | 55 | 1358 | Pacific DNA |
| PCR 2 | Ls_16S_Seq_F | Ls_16S_Rev | 55 | 764 | Pacific DNA |
| PCR 3 | Ls_16S_Fwd | Ls_16S_Seq_R | 55 | 796 | Pacific DNA |
| Seq. primers | Ls_16S_Seq_F, Ls_16s_Rev, Ls_16S_Fwd, Ls_16S_Seq_R | | | | |

Table 4: Primers used

| Primer name | Sequence | Source |
| --- | --- | --- |
| COI_seq_fwd2 | TGCAATAGTAGCAATTGGAGTATT | new |
| COI_seq_rev2 | ATGTAGTTGCCCACTTTCACTATG | new |
| COI_seq_R | AAGAGTAAAAGCACAGCAGT |  |
| LsCOI_Fwd | GAGGGTTCTTKTGAGTGT | new |
| LsCOI_Rev | GTTTCTATCTCAGGGCTTT | new |
| COI_seq_F | ACTATTACAAATTTACGGTG |  |
| Ls_16S_Rev | CGGTATAGGGATTACCCAGA | new |
| Ls_16S_Fwd | AAAGTGGCAGAGAAAGTG | new |
| Ls_16S_Seq_F | ACAAAGACATAGTAAGACTGT | new |
| Ls_16S_Seq_R | AGGTCTTAGGGTCTTATCGT | new |
|  |  |  |
|  |  |  |
|  |  |  |
|  |  |  |

1. Yazawa R, Yasuike M, Leong J, von Schalburg KR, Cooper GA, et al. (2008) EST and Mitochondrial DNA Sequences Support a Distinct Pacific Form of Salmon Louse, Lepeophtheirus salmonis. Mar Biotech 10: 741-749.
